# Bilateral Polypoid Degeneration of the Inferior Turbinates Mimicking Intranasal Neoplasia: A Case Report

**DOI:** 10.7759/cureus.89739

**Published:** 2025-08-10

**Authors:** Ourania Natsiopoulou, Dimitra Riga, Evangelos I Giotakis, Aris I Giotakis

**Affiliations:** 1 First Department of Otorhinolaryngology, Hippocration General Hospital of Athens, Medical University of Athens, National and Kapodistrian University of Athens, Athens, GRC; 2 Department of Pathology, Hippocration General Hospital of Athens, Athens, GRC; 3 Department of Otorhinolaryngology, Medical School, University of Athens, Athens, GRC; 4 Department of Otolaryngology-Head and Neck Surgery, Hippocration General Hospital of Athens, Athens, GRC; 5 Department of Otolaryngology-Head and Neck Surgery, Hippocration Hospital, University of Athens, Athens, GRC

**Keywords:** approach, degeneration, endoscopic, nasal, polyps, rhinosinusitis

## Abstract

Lesions of the inferior turbinate are exceedingly rare, with one retrospective series identifying only 34 cases over 14 years. Of those, approximately 68 % were benign (predominantly hemangiomas), and about 32 % were malignant (most commonly non-Hodgkin’s lymphoma). Data on polypoid degeneration of the inferior turbinate are limited, particularly regarding histopathologic features and treatment outcomes. This case report aims to contribute to the existing literature by providing detailed clinical, radiologic, and histologic findings, as well as therapeutic considerations for this rare entity. We report the case of a 43-year-old man from Africa (Democratic Republic of the Congo) presenting with a three-month history of severe nasal obstruction, mouth breathing, and intermittent bilateral epistaxis. Physical examination revealed large, soft, pinkish polypoid masses obstructing both nasal cavities. Imaging studies showed bilateral paraseptal tumors without paranasal sinus involvement. Endoscopic exploration revealed that the tumors originated from the medial mucosal surfaces of the inferior turbinates. The masses were excised with monopolar cautery along with a small part of the normal mucosa of the rest of the inferior turbinates. Histopathological examination revealed polypoid degeneration of fibrous tissue with inflammatory infiltration, edema, seromucous glands, and congested blood vessels. Postoperatively, the patient received antibiotics, nasal irrigation, and nasal ointment. The patient reported substantial improvement in nasal breathing one month later. Nasal cavities were patent without crusting. Polypoid degeneration of the inferior turbinate, although very rare, should be included in the differential diagnosis of inferior turbinate pathology.

## Introduction

Each nasal cavity contains three pairs of turbinates, namely the superior, the middle, and the inferior turbinates. The middle and superior turbinate are parts of the ethmoid bone, whereas the inferior turbinate is an independent bone [1]. Although studies have primarily focused on the middle turbinate, pathologies of the inferior turbinate have also been observed. While tumors of the inferior turbinate are relatively rare, they can be either benign or malignant, with benign tumors being more common. Additionally, systemic diseases can be added to the differential diagnosis, as some of them can mimic nasal tumors clinically and radiologically. Examples include granulomatosis with polyangiitis (Wegener’s granulomatosis), sarcoidosis, and other inflammatory or granulomatous diseases that may present with nasal masses or mucosal swelling resembling tumors of the inferior turbinate [2]. The reported prevalence of inferior turbinate tumors varies across studies, often depending on geographic region, population demographics, and study design. Some studies reflect global data, while others are limited to specific populations or healthcare centers.

Some of the most common benign tumors are: inverted papillomas (~0.5-4%) [2,3], osteomas (~1-3%) [2,3], hemangiomas (~4-8%) [2,3], schwannomas (~2-4%) [2,3], angiofibromas (~10-15%) [2,3]. On the other hand, malignant tumors of the inferior turbinate (30-40% of all inferior turbinate tumors) are rare and usually represent a minority of nasal cavity malignancies. The most frequent malignant types are squamous cell carcinomas (~50-70%) [2], adenocarcinomas (~10-15%) [2], melanomas (~3-4%) [2], lymphomas (~5-10%) [2] and sarcomas (~1-5%) [2]. Polypoid degeneration of the inferior turbinate is less commonly studied compared to similar changes in the middle turbinate [4,5], which is highly associated with allergic rhinitis and chronic rhinosinusitis with nasal polyps [6,7].

To date, data on polypoid degeneration of the inferior turbinate remain limited, potentially due to factors such as underreporting, diagnostic difficulty, and misclassification with more common nasal conditions. In this case report, we describe the case of a patient with bilateral polypoid degeneration of the inferior turbinate.

## Case presentation

A 43-year-old man from Africa (Democratic Republic of the Congo) presented in June 2024 to the emergency room of the First University Department of Otorhinolaryngology in Athens due to a three-month history of severe nasal obstruction, mouth breathing, and rare bilateral episodes of epistaxis. The frequency of epistaxis has increased over the past months. Therefore, the surgical management is intended to address the source of epistaxis. His medical history did not include either major pathology or surgical history. Furthermore, neither allergies nor regular medication were mentioned.

**Figure 1:**
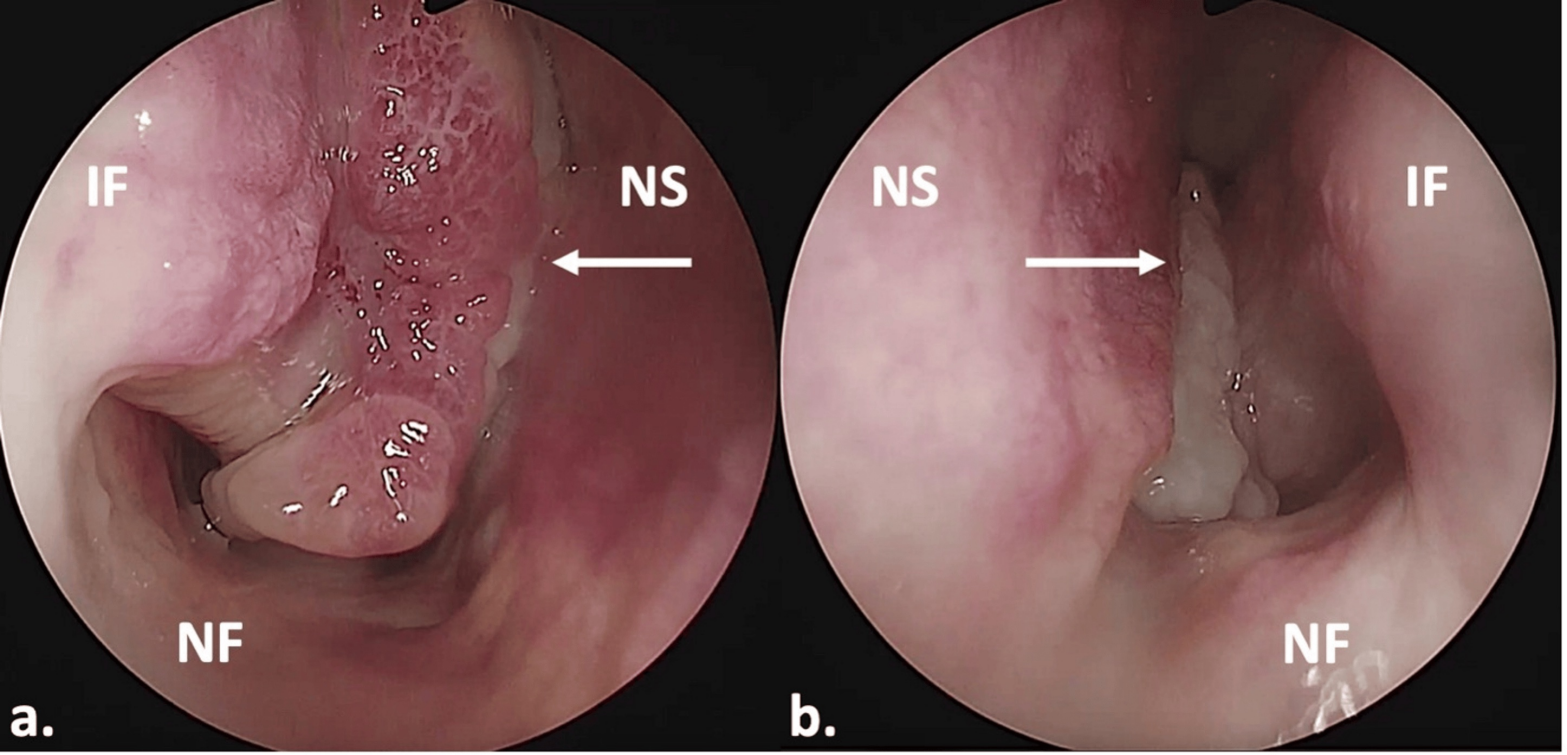
Nasal endoscopy. (a) Right side; (b) left side. The arrows pointed at the masses. IF: inferior turbinate; NS: nasal septum; NF: nasal floor.

Physical examination revealed smooth, compressive, irregular, pinkish masses obstructing both nasal cavities during anterior rhinoscopy. Nasal endoscopy revealed the presence of pinkish, large polypoid masses occupying both nasal cavities, attaching to the medial mucosal aspect of the inferior turbinates (Figure 1). The right middle meatus was visible and clear. The left middle meatus and the nasopharynx were not accessible.

**Figure 2:**
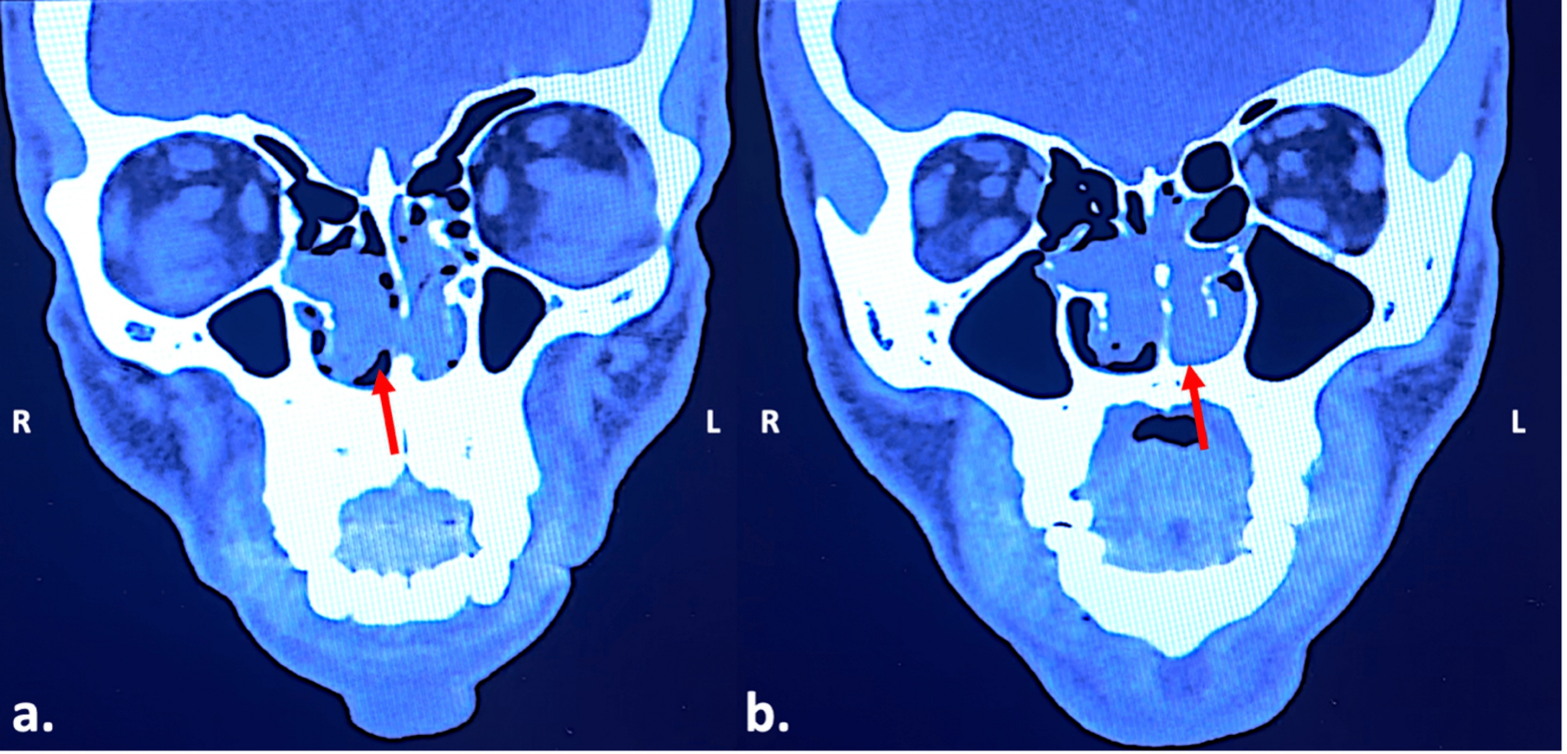
Computed tomography scan of the head without contrast medium (coronal view) revealed gray instead of black area (indicated by the red arrows) medial to the inferior turbinates. The paranasal sinuses were filled with air. (a) Anteriorly; (b) posteriorly; R: right; L: left.

**Figure 3:**
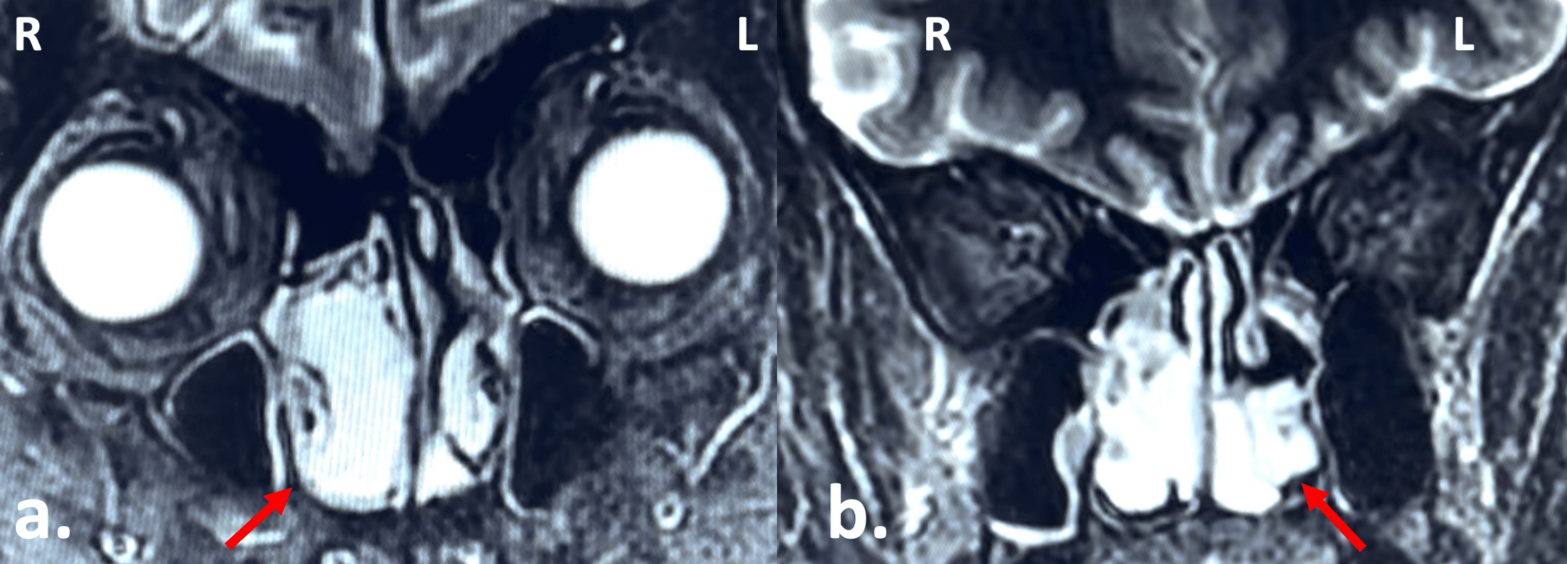
Magnetic resonance imaging of the head with gadolinium (T2-sequence; coronal view) revealed similar enhancement of the pathologic tissue (indicated by the red arrows) compared to that of the inferior turbinates. (a) Anteriorly; (b) posteriorly; R: right; L: left.

Blood tests, including complete blood count, metabolic tests, liver and renal function, as well as electrocardiography and chest X-ray, were normal. A computed tomography of the head without contrast medium and a magnetic tomography of the head with gadolinium revealed a paraseptal mass located in both nasal sides without any involvement of the paranasal sinuses, the orbit, or the skull base (Figures 2, 3).

**Figure 4:**
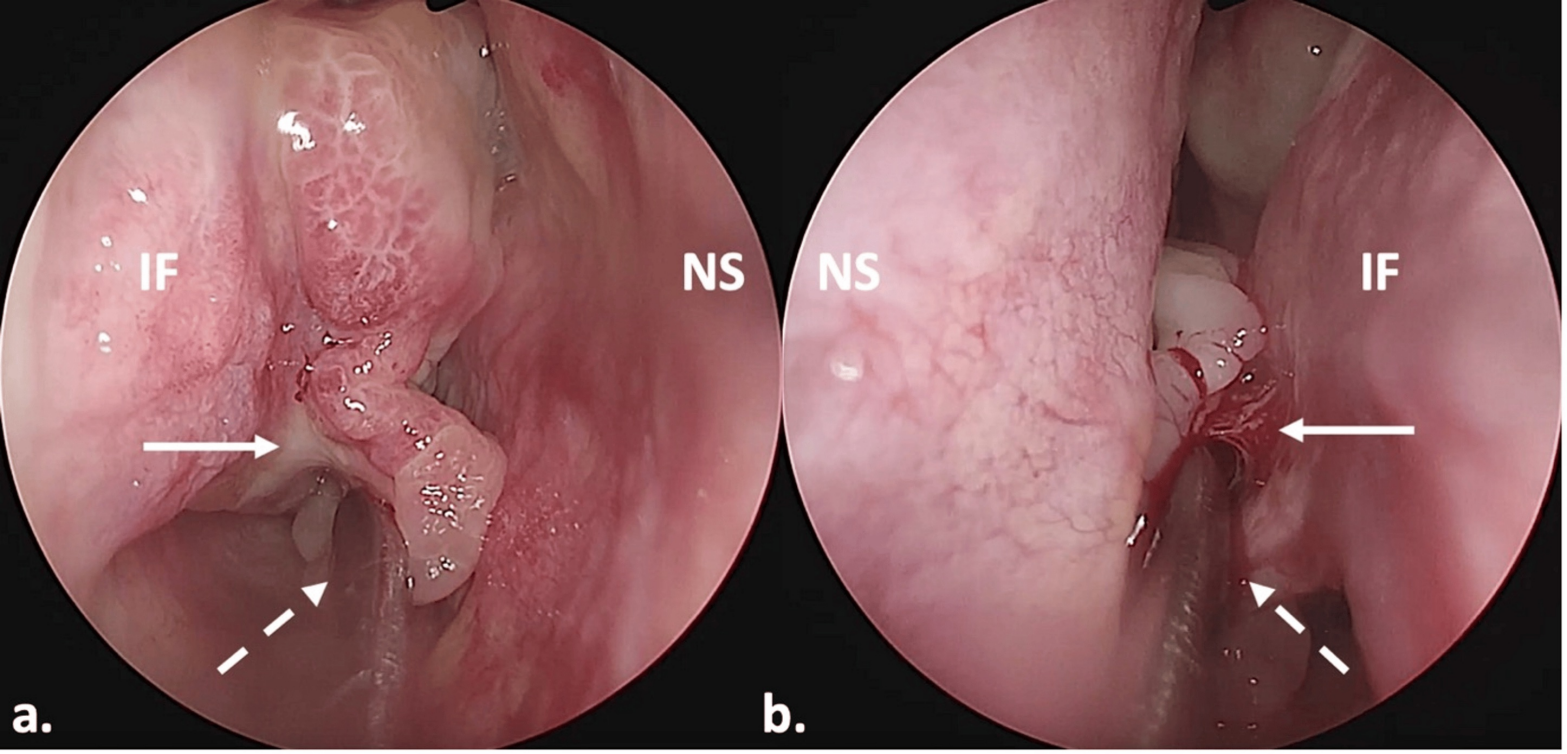
Intraoperative finding. (a) Right side; (b) left side. Pathologic tissue displaced medially by a surgical freer (indicated by the dashed arrows). The arrows pointed at the attachments of the inferior turbinates. IF: inferior turbinate; NS: nasal septum; NF: nasal floor.

**Figure 5:**
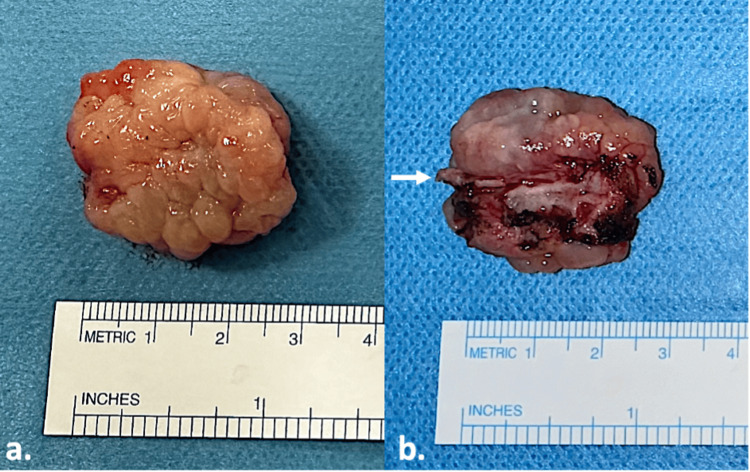
Excised right specimen. (a) Direct view of the specimen (medial view). (b) Specimen turned upside-down (lateral view). The arrow pointed at the turbinate bone attachment. Small amount of normal turbinate mucosa around the bone. Metric in centimeters.

Due to the risk of obtaining a non-representative tissue sample with office-based incisional biopsy [8], the patient was scheduled for endoscopic surgical exploration. This approach was further justified by the findings of the study Clinical Value of Office-Based Endoscopic Incisional Biopsy in Diagnosis of Nasal Cavity Masses, which reported that office-based biopsies provided an accurate histopathological diagnosis in only 44% of cases. The limited diagnostic yield is often attributed to superficial sampling, inadequate visualization, or sampling from necrotic or non-diagnostic areas of the lesion. In contrast, endoscopic surgical exploration allows for enhanced visualization and access, enabling the collection of deeper and more representative tissue specimens, which is particularly important when malignancy or uncommon pathology is suspected. This would involve precise demarcation of the tumor and its attachment sites, as well as tumor excision if possible. Under general anesthesia, he was placed on the operating table in reverse Trendelenburg, with the head elevated about 30 degrees and turned slightly to the right. Both nasal cavities were packed with multiple neurosurgical patties soaked in a solution of 5 ml of adrenaline in 20 ml of NaCl 0.9% for decongestion. A thorough nasal endoscopy was performed using a 0-degree scope. With the help of a freer elevator, we found that the masses originated from the medial surface of each inferior turbinate (Figure 4). Excision of the masses was performed using sharp surgical technique (Figure 5) with the help of a 45° angled-down monopolar microdissection electrode with a tip and a total length of 229 mm (ARROWtip™ monopolar microdissection electrode Basterra 45° angled down by Sutter Medizintechnik GmbH, Emmendingen, Germany). A part of the inferior turbinates was preserved. Nasal tamponades were put in both nasal cavities.

**Figure 6:**
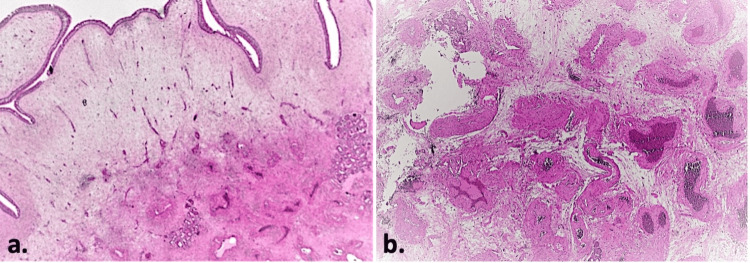
Histopathologic examination (hematoxylin-eosin stain, 10X). (a). Left nose: sinonasal polypoid lesion, covered by respiratory-type epithelium, surrounding edematous and hemorrhagic stroma, with vascular channels and seromucinous glands. (b) Right nose: edematous stroma, with scattered erythrocytes and thick-walled, hyperplastic, congested and hyperemic vessels, along with mild to moderate chronic inflammation.

**Figure 7:**
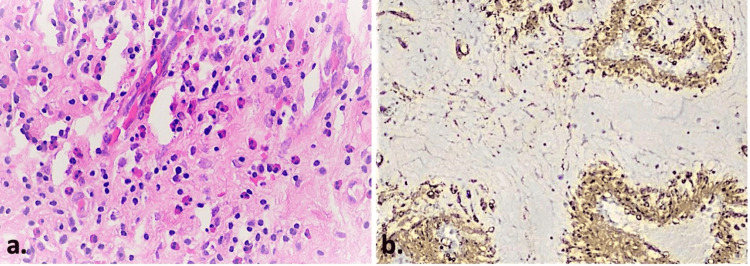
Histopathologic examination, higher magnification, and immunohistochemistry. (a) The number of polymorphonuclear eosinophils exceeded 25 eosinophils/high-power-field (hematoxylin-eosin stain, 40X). (b) Smooth muscle actin (SMA) immunostain expressed in the hyperplastic smooth muscle fibers of the vascular walls as well as in the stromal fibroblasts (SMA immunostain, 10X).

The excised tissues were sent to the Department of Pathology for histopathological examination. The results of the examination reported polypoid transformation of fibrous tissue, which was lined by respiratory epithelium and enclosed edema, hemorrhagic infiltrates, seromucous glands, and moderately dense inflammatory infiltration with involvement of lymphocytes, plasma cells, and eosinophils. Within the fibrous tissue, smooth muscle fibers coexist, among which congested blood vessels were present. The pathology report indicated bilateral polypoid degeneration of the inferior turbinates (final sign-out) (Figures 6, 7).

The patient received postoperatively ampicillin and sulbactam 3g every eight hours and paracetamol if needed. The Merocel nasal packs were removed on the first postoperative day. The patient was discharged on that day in good condition. The postoperative management included nasal irrigation with normal saline and application of nasal moisturizing cream (Rinopanteina; PharmaQ, Athens, Greece) three times daily for a month.

Approximately a month after surgery, the patient reported significant improvement in nasal breathing. He noted no epistaxis. Physical examination revealed patent nasal cavities without crusting.

## Discussion

This case may be the first reported instance of bilateral polypoid degeneration of the inferior turbinate. The existing literature on polypoid degeneration of the inferior turbinate is sparse, with very few publications addressing this entity directly, and only one study was identified as partially relevant [9]. This highlights a notable gap in the current understanding of this rare condition. Hyperplasia of the posterior aspect of the inferior turbinate is a common finding in allergic rhinitis and is typically not mistaken for a neoplastic process [10].

On the contrary, we described here the case of a patient, in which the clinical diagnosis by an otolaryngologist was nasal tumor. Contrary to expectations, this case was initially diagnosed as a nasal tumor, although such tumors are a rare cause of progressive bilateral nasal obstruction. However, in this 43-year-old patient, the presence of intermittent epistaxis accompanied by soft, pink-gray papillary masses with a convoluted or wrinkled surface raised suspicion of a neoplastic lesion.

The bilateral occurrence of these masses posed a diagnostic challenge. However, synchronous bilateral nasal tumors have been described previously [11-15]. Furthermore, the absence of involvement of the paranasal sinuses in CT and MRI might have pointed towards a midline nasal tumor [16-19]. Moreover, the friability of this tissue, combined with its tendency to bleed, did not allow us to examine it properly under local anesthesia.

Therefore, we aimed to carry out a surgical mapping with several biopsies. Alternatively, we could have taken a biopsy under local anesthesia. However, the risk of a non-representative result might have been high. According to Han and coauthors, non-representative biopsies may reduce sensitivity to 44% in paranasal sinus lesions [8].

Unexpectedly, exploration under general anesthesia revealed two independent masses originating from the medial surface of the inferior turbinates. Again, considering the risk of a non-representative biopsy, we resected the masses completely with close margins. The purpose of performing these excisional biopsies was to give pathologists ample tissue for diagnostic assessment. Furthermore, this decision was based on the possible resectability of these masses without any potential functional deficits.

After discharge on the first postoperative day, the patient returned for a planned follow-up one month later. At that time, both nasal cavities were patent and free of crusting. Unfortunately, the patient was subsequently lost to follow-up and did not respond to further attempts at communication. As a result, long-term assessment-including evaluation of recurrence or residual symptoms-was not possible, representing a limitation in our ability to fully assess the outcome of the intervention. 

Nevertheless, excisional biopsy of the symptomatic polypoid degeneration of the inferior turbinate that was mimicking bilateral nasal tumors seemed sufficient for diagnosis and treatment. A comparison to other treatments of polypoid degeneration of the inferior turbinate was not possible due to a lack of similar studies.

## Conclusions

Polypoid degeneration of the inferior turbinate, although a very rare pathology, may be considered in the differential diagnosis of inferior turbinate lesions. En bloc excisional biopsy of the masses, including a small portion of the inferior turbinates and their attachment sites, may represent an effective treatment option in selected cases. However, given the rarity of the condition and the limited duration of follow-up in this case, further studies are needed to validate this approach.
